# Narrative Review of Genetic and Immunological Mechanisms Involved in the Pathogenesis of Kimura’s Disease: New Therapeutic Targets

**DOI:** 10.3390/genes16020194

**Published:** 2025-02-04

**Authors:** Antonella Loperfido, Carlo Cavaliere, Bruno Fionda, Gianluca Bellocchi, Simonetta Masieri, Marco Caminati

**Affiliations:** 1 Otolaryngology Unit, San Camillo Forlanini Hospital, Circonvallazione Gianicolense 87, 00152 Rome, Italy; 2 Department of Sense Organs, Sapienza University, Piazzale Aldo Moro 5, 00185 Rome, Italy; 3 Fondazione Policlinico Universitario Agostino Gemelli IRCCS, 00168 Rome, Italy; 4 Department of Oral and Maxillofacial Sciences, Sapienza University, Piazzale Aldo Moro 5, 00185 Rome, Italy; 5 Allergy Unit and Asthma Center, Verona Integrated University Hospital, 37134 Verona, Italy; 6 Department of Medicine, University of Verona, 37124 Verona, Italy

**Keywords:** Kimura’s disease, biologics, biological therapies, eosinophils, type 2 inflammation

## Abstract

Kimura’s disease (KD) is a rare, chronic inflammatory disorder that predominantly affects young men of East Asian descent. It is characterized by painless solid masses primarily localized to the deep subcutaneous tissues of the head and neck, eosinophilia, and elevated serum immunoglobulin E (IgE). While the exact cause remains unclear, the pathogenesis is thought to involve dysregulated immune responses, particularly those mediated by T-helper cells 2 (Th2), eosinophils, and IgE production. Advances in molecular biology have suggested that genetic factors play a significant role in the development and progression of this chronic inflammatory condition. Recent studies have implicated several genes and immune pathways in its development, and understanding these genetic components may provide insights into better diagnostic tools and therapeutic strategies for KD. In this regard, biological therapies, by targeting the immune mechanisms underlying KD, have been used to treat this challenging condition with promising results, contributing to a better understanding of the pathogenesis of this rare disorder. The aim of this study was to review the literature concerning the genetic factors and immune mechanisms that contribute to the pathogenesis of KD, with a special focus on the role of biological therapies.

## 1. Introduction

Kimura’s disease (KD) is an idiopathic condition that presents as a chronic, benign inflammatory disorder, typically affecting the head and neck region. The disease was first described in the scientific literature in 1937 by Chinese researchers, who initially referred to it as “eosinophilic hyperplastic lymphogranuloma” [1]. Later, the condition was defined as KD in 1948, when a Japanese research group published its definitive histopathological features [2]. Clinically, this condition typically presents as painless, solid masses, primarily localized to the deep subcutaneous tissues of the head and neck, with a predilection for the salivary glands, particularly the parotid gland, and adjacent lymph nodes [3,4]. The histological findings demonstrate the presence of proliferating blood vessels and eosinophilic infiltration, indicating an inflammatory response potentially linked to a hypersensitivity or autoimmune reaction triggered by an unidentified trigger [5]. A history of atopy is frequently identified. In some instances, the disease may be associated with prurigo or impact renal function, leading to nephrotic syndrome [6,7,8]. Furthermore, some studies have also reported the association with ulcerative colitis, cardiovascular disease, hepatitis, and asthma [9,10,11,12]. Along with the presence of masses in the head and neck, the most prominent features of this disease include elevated serum levels of immunoglobulin E (IgE) and increased peripheral blood eosinophil count [13,14]. The condition primarily affects young Asian males, typically between the ages of 20 and 50, with a male-to-female ratio ranging from 3.5:1 to 9:1 [15]. Although no cases of malignant transformation have been reported to date, active treatment is required, as spontaneous resolution has been documented in rare cases [16]. Additionally, this condition is typically prone to relapse [17].

For the management of this condition, a range of therapeutic approaches are described in the literature, such as corticosteroids, cyclosporine, and leflunomide. However, the limitations of these standard pharmacological treatments are well documented, including the possible side effects of prolonged oral corticosteroid use, such as diabetes, weight gain, facial swelling, infections, anxiety, insomnia, hyperactivity, mood swings, and reduced bone mineral density. On the other hand, long-term side effects of cyclosporine include kidney injury and increased risk of infection. More recently, monoclonal antibodies developed to target T2-high inflammation, such as Dupilumab, Mepolizumab, and Omalizumab, have been introduced in management and serve as a potential solution [18,19,20,21,22,23,24]. Furthermore, surgical excision and radiotherapy are commonly considered effective treatment options [25]. However, no definitive clinical guidelines are currently available to standardize their application.

While KD etiology is largely unknown, it is generally considered to result from an aberrant immune response. Recent studies have implicated several genes and immune pathways in its development, and understanding these genetic components may provide insights into better diagnostic tools and therapeutic strategies for KD.

## 2. Pathogenesis of Kimura’s Disease: Genetic and Immunologic Mechanisms

KD is often classified as a so-called type 2 inflammatory disorder in the light of its immunological background characterized by an over-expression of T helper 2 (Th2) driven pathways, including an increased production of T2 cytokines and eosinophil activation [26]. Indeed, elevated levels of IgE, tumor necrosis factor α (TNF-α), interleukin (IL)-4, IL-5, and IL-13, as well as an increase in mast cells and eosinophils, have been observed in the majority of patients [27,28]. Furthermore, immunohistochemical analysis of KD lesions revealed increased infiltration of mast cells, activated eosinophils, and T cells, as well as IL-4+, IL-5+, eotaxin+, RANTES+, and CCR3+ cells [29], thereby supporting the predominance of a Th2-mediated response. In this regard, biopsy of lesions is considered the gold standard for the diagnosis of KD, as the reactive nature of this chronic inflammatory condition is demonstrated by characteristic histopathological features, including marked follicular hyperplasia, inter- and intra-follicular prominent eosinophilic and lymphoplasmacytic infiltration, increased small blood vessels, eosinophilic micro-abscesses (with or without Charcot-Leyden crystals), and dense stromal fibrosis [30]. In addition, the normal germinal centers may be replaced by IgE or eosinophilic deposits, necrotic tissue, and the presence of polykaryocytes, such as Warthin–Finkeldey-type giant cells [31,32]. Some authors have also reported that, in certain patients, inflammatory infiltration of nerve fibers by lymphocytes and eosinophils can lead to pruritus [33].

However, despite extensive research, the etiology and pathogenesis of KD remain undefined, as no specific antigens activating the immune cascade have been identified [34]. Allergic/hypersensitivity reactions, neoplasms, infections, and autoimmune responses with an aberrant immune reaction have been proposed as potential etiological mechanisms [35,36].

Similarly to other T2 conditions, it has been hypothesized that both genetic susceptibility and environmental factors contribute to the pathogenesis of the disease; in fact, several genes have been identified as a predisposing condition to the underlying inflammatory processes.

### 2.1. Immune-Related Genes

#### 2.1.1. T-Cell Receptor and Th2 Cytokines

One of the primary immune responses implicated in KD is the overactivation of Th2 cells, which release specific cytokines, including IL-4, IL-5, and IL-13. These cytokines play a critical role in driving eosinophil differentiation and activation, as well as in promoting IgE production, all of which are hallmark features of the disease [37].

Specifically, in the pathogenesis of eosinophilia, cytokines such as IL-5, granulocyte-macrophage colony-stimulating factor (GM-CSF), and IL-3 are key mediators, promoting eosinophilic infiltration and facilitating the prolonged survival of eosinophils [38]. Chemokines, such as eotaxin and RANTES, are critical in mediating the recruitment of eosinophils to sites of inflammation, thereby contributing to localized eosinophilic infiltration. Furthermore, as already mentioned, IL-4 and IL-13 represent important cytokines that participate in the induction of IgE synthesis [39]. Some studies have documented the expression and/or secretion of cytokines such as IL-4, IL-5, and GM-CSF by peripheral blood mononuclear cells in KD [40].

Interestingly, genetic polymorphisms in the IL4 and IL5 genes have been associated with increased Th2 responses and eosinophilic inflammation in several allergic and inflammatory diseases, suggesting a potential role in KD [41].

The T-cell receptor (TCR) complex and co-stimulatory molecules are essential for initiating the Th2 immune response [42]. Alterations in TCR signaling could result in excessive activation of CD4+ T-cells, driving the inflammatory cascade in KD [43]. Thus, any genetic variation that alters T-cell signaling might predispose individuals to an amplified immune response, potentially contributing to the disease’s pathogenesis.

#### 2.1.2. Interleukin-4 (IL-4), Interleukin-5 (IL-5), and Interleukin-13 (IL-13)

Several studies have demonstrated that Th2 cytokines, including IL-4, IL-5, and IL-13, are abundantly produced in KD patients [29,44].

IL-4 is a key cytokine that drives the differentiation of naïve T-cells into Th2 cells. It also stimulates B cells to produce IgE. The IL4R gene, which encodes the IL-4 receptor, has been implicated in the pathogenesis of other Th2-related diseases, such as asthma, allergic rhinitis, and atopic dermatitis [45], and may similarly contribute to KD through elevated IL-4 signaling. In this regard, a previous study has demonstrated that IL-4-producing cells were abundant in affected tissues from KD patients [29]. Moreover, a recent study has demonstrated the over-activation of extracellular-signal-regulate kinase/mitogen-activated protein kinases (Erk/MAPK) signaling pathway in eosinophils associated with KD, which is precisely a pathway involved in the induction of IL-4 and IL-13 production [46,47].

Concerning IL-5, this cytokine plays a central role in the differentiation, recruitment, survival, activation, and degranulation of eosinophils [48], which are abundant in KD lesions. Increased IL-5 expression, potentially driven by genetic polymorphisms, may thus contribute to the eosinophilic infiltration seen in this inflammatory condition [49].

Regarding research on IL-13, Munemura et al. recently demonstrated that infiltrating IL-13-expressing Tfh cells and type 2 immune cells are abundant in tissue lesions of patients with KD. Furthermore, these authors also highlighted that those patients exhibited an increased eosinophil count and elevated IgE levels [50].

### 2.2. Eosinophil Activation and Recruitment

Eosinophils are critical effector cells in KD. Two main eosinophil-mediated pathobiological mechanisms can be identified in the background, including inflammation and direct tissue damage. The first is related to the relevance of eosinophils within the T2 immunological frame, as they are the target and driver of cells and cytokines typically implicated in the activation, amplification, and chronic evolution of that inflammation type. In addition, eosinophils do contain in their cytoplasm a number of proteins and enzymes, and eosinophilic cationic protein (ECP) is the most relevant one, which is able to exert direct tissue damage [51,52]. As the pathobiological and clinical counterpart of the mentioned mechanisms, the literature describes that eosinophilic microabscesses and eosinophil-infiltrated neural fibers are characteristic features in affected patients [50]. Interestingly, a recent study found that an increased number of Group 2 innate lymphoid cells (ILC2s) in the blood was associated with blood eosinophilia, elevated IgE levels, and pruritus in KD patients [53]. ILC2s have been recognized as key effector cells in eosinophilic airway inflammation, including conditions such as asthma, allergic rhinitis, and chronic rhinosinusitis [54].

Several genes involved in eosinophil recruitment and activation have been implicated in KD.

#### 2.2.1. Eosinophil Cationic Protein (ECP)

ECP is a cytotoxic protein released by eosinophils that contributes to tissue damage during inflammation. Elevated ECP levels are often seen in KD patients, suggesting its role in the disease’s pathophysiology [55]. Genetic variation in ECP or related pathways could affect the severity of eosinophil-driven inflammation.

#### 2.2.2. CCR3 and Eotaxins

The chemokine receptor CCR3 and its ligands, particularly eotaxin-1 (CCL11), play pivotal roles in the recruitment of eosinophils to sites of inflammation. Increased expression of CCR3 and eotaxin-1 in KD lesions may enhance eosinophil migration and contribute to tissue damage [29]. Studies suggest that polymorphisms in the CCR3 gene could increase susceptibility to conditions involving eosinophilic inflammation [56], including KD.

### 2.3. Immune Regulation and T Regulatory Cells (Tregs)

Regulatory T cells (Tregs) play a crucial role in maintaining immune tolerance by suppressing excessive or auto-reactive immune activation [57]. Dysregulation of Tregs has been noted in several autoimmune and inflammatory diseases, including those resulting from allergic, autoimmune, or infectious causes [58,59]. The FOXP3 gene, which is critical for Treg development, may be involved in the abnormal immune responses seen in KD. Mutations or polymorphisms in FOXP3 could impair Treg function, leading to a failure in suppressing the inflammatory response and contributing to disease progression [60].

### 2.4. Immunologic Mechanisms Leading to Fibrosis

Lesions associated with KD are typically characterized by the presence of fibrosis. Specifically, fibrosis represents a common final outcome of most chronic inflammatory conditions triggered by a variety of stimuli, such as autoimmune reactions, allergic responses, persistent infections and exposure to various forms of tissue injury. In this context, type 2 immune cells, including eosinophils, and associated cytokines such as IL-4, IL-5, and IL-13 play a crucial role in the pathogenesis of allergic inflammation and fibrosis. Type 2 immune response stimulates a complex inflammatory reaction involving mast cells, eosinophils, basophils, Th2 cells, type 2 innate lymphoid cells, and specific subclasses of IgE antibodies, all of which potentially contribute to inflammation chronicity and to the pathogenesis of fibrotic evolution. In particular, eosinophils have been associated with the progression of lung and skin fibrosis in chronic asthma and atopic dermatitis [61,62].

In fact, several eosinophil-derived granule proteins and pro-fibrotic cytokines play a crucial role in these processes. Among these, Galectin-10 (also known as Charcot–Leyden crystal protein), which is released by activated eosinophils during type 2 immune responses, serves as a marker of eosinophil death and can persist in tissues for months [63]. It has been observed that Galectin-10-positive cells are expanded in the lesions of patients with KD [50]. Another relevant protein is Osteopontin (OPN), an extracellular matrix protein linked to fibrotic disorders and implicated in allergic diseases. Studies have shown that OPN-positive eosinophils are abundant in KD patients [50]. Lastly, Amphiregulin has been found to reprogram the eosinophil transcriptome toward an inflammatory state, promoting the secretion of OPN [64]. Amphiregulin (AREG)+CD4+ T cells have been detected and are abundant in affected tissues of patients with KD [50].

Therefore, it is plausible to hypothesize that the specific subset of recruited eosinophils may represent the primary inflammatory cell population responsible for driving TH2 cell-mediated fibrosis [65].

Table 1 summarizes genetic and immunologic mechanisms involved in the pathogenesis of KD.

## 3. Gene-Environment Interactions

In addition to genetic susceptibility, environmental factors likely contribute to the onset of KD, including a smoking habit and systemic diseases (for example, hepatitis B, hepatitis C, hypertension, cardiovascular disease, asthma, and nephrotic syndrome). Allergens and chronic infections have been proposed as potential environmental determinants recurrently or chronically triggering an impaired immune response in predisposed individuals. These factors may interact with the host’s genetic predisposition, initiating the inflammatory cascade characteristic of the disease. Although the etiology of KD remains unclear, it is thought to involve disruption or dysregulation of immune responses, potentially triggered by an atopic reaction to chronic antigenic stimuli, such as viral infections, arthropod bites, or neoplasms. One intriguing hypothesis suggests that Candida may serve as a persistent source of antigenemia; however, hyphae and spores have not been successfully isolated from affected individuals [7].

## 4. Immunologic Targeted Treatment: Biological Therapies

**Figure 1:**
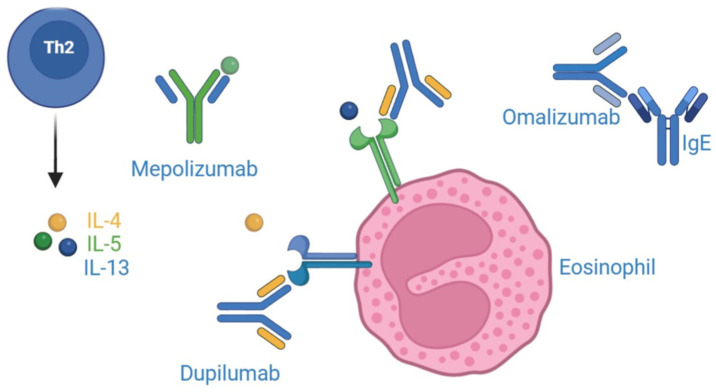
Mechanisms of action of the main biologics used in Kimura’s disease.

T2 inflammation has been first explored as a potential target of selective treatments in the field of severe asthma. Different monoclonal antibodies addressing specific drivers of the immune response, including IL-5, IL-4, IL-13, IgE, and thymic stromal lymphopoietin (TSLP), are currently licensed for that indication, and an increasing amount of evidence supports their steroid-sparing effect and their ability to interfere with the disease evolution [66]. That background has paved the way for the investigation of anti-T2 monoclonal antibodies in different conditions other than asthma but sharing common immunological features typically characterizing T2 inflammation [67,68]. This is the case of KD; in fact, in the light of its typical features, including elevated serum IgE levels, peripheral blood eosinophilia, and eosinophil infiltration in tissues, a Th2 cell-mediated immune response has been proposed, and the recent findings linking KD to IgG4-related diseases further support this hypothesis [69]. Consequently, various biologic therapies, including anti-IL-4/IL-13, anti-IgE, and anti-IL-5, have been used to treat KD with promising results, contributing to a better understanding of the pathogenesis of this rare disorder. In addition, no ongoing clinical trial has been officially registered specifically for KD. In Figure 1, we report a summary of the mechanism of action of the main biologics included in the present review.

### 4.1. Role of Anti-IL-4/IL-13 Receptor (Dupilumab) in KD

Since Dupilumab is a fully human-derived monoclonal antibody that specifically targets IL-4Rα, thereby inhibiting IL-4 and IL-13 signaling, it has been approved for the treatment of several diseases involving the Th2 pathway, including atopic dermatitis, asthma, eosinophilic esophagitis, chronic rhinosinusitis with nasal polyps, and prurigo nodularis. Its safety has been well documented [70,71]. Based on the involvement of the Th2 pathway in KD, there has been an increasing number of clinical reports in the literature regarding its use in managing this challenging condition.

In this regard, Liu et al. recently reported on six KD patients treated with Dupilumab, showing a significant reduction in serum total IgE levels, as the biologic targets IL-4 and IL-13, cytokines known to typically promote IgE antibody production. Furthermore, the authors highlighted that Dupilumab effectively reduced the nodules commonly associated with the disease, as well as eosinophil counts and percentages, leading to clinical improvements in these six patients [72].

Another recent report by Lyu et al. describes a woman diagnosed with KD who presented with atopic dermatitis, multiple lymphadenopathies, and limb swelling. She was treated with Dupilumab alongside concurrent oral corticosteroids. Following treatment, the multiple nodules showed a significant reduction in size, while serum IgE levels, eosinophil, and basophil counts all decreased substantially. These findings highlight the considerable effectiveness of combining the biologic agent with oral corticosteroids in managing KD in patients with concomitant atopic dermatitis [73].

Yang et al. reported a case of a 57-year-old male diagnosed with KD who was treated with Dupilumab receiving an initial dose of 600 mg, followed by 300 mg every two weeks for four months. Treatment resulted in a reduction of mass size and a rapid decrease in eosinophil counts, although serum IgE levels remained unchanged [22].

Huang et al. reported the use of Dupilumab following surgical intervention in a 36-year-old male patient with KD, who presented with an enlarging mass in the left medial thigh and chronic eczema on the abdomen and lower legs. The authors administered Dupilumab postoperatively, starting with an initial dose of 600 mg, followed by 300 mg every two weeks for 8 months. The patient achieved complete relief, with no recurrence of KD observed during a 1-year follow-up and significant improvement in the eczematous lesions [74].

Also, Teraki et al. [75] and Suga et al. [76] reported the efficacy of Dupilumab in the management of KD, utilizing a 600 mg loading dose followed by 300 mg every two weeks, with follow-up periods of 10 and 15 months, respectively.

Bellinato et al. reported a case of a 59-year-old patient with refractory KD who was successfully treated with Dupilumab, administered subcutaneously at a dose of 300 mg every other week. After 6 months of follow-up, no recurrence was observed [77].

Shang et al. described a pediatric case involving a 14-year-old boy diagnosed with KD who presented with recurrent enlargement of the left retroauricular and cervical lymph nodes despite surgical treatment. The patient also exhibited nephrotic syndrome, persistently elevated IgE levels, and fluctuating eosinophil counts. The patient was treated with Dupilumab at a loading dose of 600 mg, followed by a maintenance dose of 300 mg every two weeks. This treatment resulted in a decrease in IgE levels, stabilization of eosinophil counts, and a reduction in the size of the lymph nodes [37].

Finally, Luo et al. recently reported on a 25-year-old female patient with KD who responded favorably to treatment with Dupilumab, administered at a dosage of 300 mg every two weeks, following an initial dose of 600 mg. The treatment was well tolerated by the patient. The authors suggested that this biologic agent could represent a promising therapeutic option for KD. However, they emphasized the necessity for larger, randomized controlled trials to validate their findings [47].

### 4.2. Role of Anti-IgE Monoclonal Antibody (Omalizumab) in KD

Regarding the efficacy of anti-IgE biologic (Omalizumab) in the management of KD, Nonaka et al. reported the first trial in 2014, treating three Japanese patients with KD that were uncontrolled by surgery. These patients were managed using a fixed schedule of eight cycles of Omalizumab 300 mg, administered subcutaneously every two weeks. The treatment led to successful outcomes, including a reduction in the size of the tumorous region and decreased peripheral blood basophil and eosinophil counts [13].

Ao et al. also provide evidence for the use of anti-Ig E therapy in the treatment of KD, reporting two cases of refractory KD that responded to a low dose of steroids combined with Omalizumab [24].

### 4.3. Role of Anti-IL5 Agents (Mepolizumab and Benralizumab) in KD

Anti-IL-5 biologics appear to be a promising therapeutic approach for KD, as it may inhibit the production, survival, and recruitment of eosinophils in both the tissues and blood. In this regard, there are several studies evaluating the treatment of KD with Mepolizumab, which targets IL-5 itself, and Benralizumab, which targets the surface IL-5 receptor (IL-5R-α).

Kinoshita et al. reported the efficacy of monotherapy with 300 mg of Mepolizumab administered subcutaneously once a month for 8 months in a KD patient by reducing the size of the mass together with the number of eosinophils. The authors also observed that, despite the disappearance of eosinophils in the mass, fibrosis persisted. This may be attributable to the patient’s 6-year history of chronic KD prior to the administration of Mepolizumab, with prolonged local inflammation potentially leading to persistent fibrosis. Conversely, the authors hypothesize that earlier intervention with Mepolizumab could have changed the outcome and prevented secondary fibrosis caused by this chronic inflammatory condition [23].

Al Shammari et al. reported a case involving a 27-year-old male diagnosed with KD associated with ulcerative colitis and successfully treated with Mepolizumab [78].

Ho et al. documented a case involving a 26-year-old male diagnosed with eosinophilic chronic rhinosinusitis and concurrent KD. The patient received 300 mg of Mepolizumab every four weeks, resulting in well-controlled symptoms during long-term therapy [79].

On the other hand, Szeto et al. demonstrated that Benralizumab also seems to produce a prolonged effect on clinical improvement in KD patients [80].

### 4.4. Role of Anti-CD20 (Rituximab) in KD

Rituximab is not part of the anti-T2 monoclonal antibodies armamentarium; in fact, it binds a specific B-cell surface antigen, namely CD 20. Its selective action on B-cells, on the one hand, provides the rationale for its use in KD; on the other, it implies an immunosuppressive effect which, differently from the molecules described above, might restrict its use in patients with chronic infections or malignancies and limit its long-term use. It is currently indicated for the treatment of non-Hodgkin lymphoma, chronic lymphatic leukemia, rheumatoid arthritis, eosinophilic granulomatosis with polyangiitis, and pemphigus vulgaris [81].

Vissing-Uhre et al. report the effects of Rituximab treatment in a patient with KD and concomitant membranous nephropathy. During 30 months of follow-up, no signs of relapse were observed, and the patient demonstrated sustained remission of nephrosis and normalization of peripheral eosinophilia [82].

In this regard, Ghosn et al. also present a case of a 37-year-old Lebanese woman with a concurrent diagnosis of KD and mycosis fungoides who was treated with Rituximab [83].

Table 2 summarizes the published experiences on the use of biologic drugs in the treatment of KD.

## 5. Conclusions

The pathogenesis of KD is multifactorial, involving complex interactions between genetic factors and immune dysregulation. The overactivation of the T2 immune response, combined with the enhanced recruitment of eosinophils and the dysregulation of immune tolerance mechanisms, contributes to the chronic inflammation and tissue damage observed in KD. Biologic therapies selectively targeting the T2 molecules implicated in the impaired immune mechanisms underlying KD may represent safe and effective treatment options for the long-term management of this complex condition. Further research into the genetic components of this rare and challenging disease will help identify potential biomarkers for diagnosis and therapeutic targets for more effective and personalized treatments.

## Figures and Tables

**Table 1:** Genetic and immunologic mechanisms involved in the pathogenesis of KD.

Etiological Pathways	Molecules	Reference
TCR and Th2 Cytokines	TCR	[43]
	IL-4	[29]
	IL-5	[49]
	IL-13	[50]
Eosinophil Activation and Recruitment	ECP	[53]
	CCR3	[54]
	Eotaxins	
Immune Regulation and Tregs	Tregs	[58]
	FOXP3	
Immunologic Mechanisms leading to Fibrosis	Galectin-10	[50]
	OPN	
	AREG	

TCR: T-cell receptor; IL: Interleukin; ECP: Eosinophil Cationic Protein; CCR3: C-C Motif Chemokine Receptor 3; Tregs: T Regulatory Cells; FOXP3: Forkhead Box P3; OPN: Osteopontin; AREG: Amphiregulin.

**Table 2:** Summary of published experiences on the use of biologic drugs in the treatment of KD.

Biologic	Authors	Year	Country	Sample Size	Gender	Age (Mean)	Site of the Masses
Dupilumab	Liu et al. [72]	2024	China	6	Male	25	H&N
	Lyu et al. [73]	2024	China	1	Female	37	H&N, Trunk, Limbs
	Yang et al. [22]	2022	China	1	Male	57	H&N
	Huang et al. [74]	2022	Taiwan	1	Male	36	Thigh
	Teraki et al. [75]	2022	Japan	1	Male	57	Arm
	Suga et al. [76]	2023	Japan	1	Male	65	H&N, Back
	Bellinato et al. [77]	2022	Italy	1	Male	59	H
	Shang et al. [37]	2023	Taiwan	1	Male	14	H&N
	Luo et al. [47]	2024	China	1	Female	25	H
Omalizumab	Nonaka et al. [13]	2014	Japan	3	2 M, 1 F	47	H&N
	Ao et al. [24]	2024	China	2	Male	21	H&N
Mepolizumab	Kinoshita et al. [23]	2021	Japan	1	Female	42	Upper Limbs
	Al Shammari et al. [78]	2019	Saudi Arabia	1	Male	27	H&N
	Ho et al. [79]	2021	Australia	1	Male	26	H&N
Benralizumab	Szeto et al. [80]	2022	Canada	1	Female	41	H&N
Rituximab	Vissing-Uhre et al. [82]	2021	Denmark	1	Male	30	H&N, Back
	Ghosn et al. [83]	2009	Lebanon	1	Female	37	Abdomen, Back

H&N: Head and Neck; M: Male; F: Female.

## Data Availability

No new data were created or analyzed in this study. Data sharing is not applicable to this article.

## Abbreviations

KD.	Kimura’s Disease
Ig	Immunoglobulin
Th2	T helper 2
TNF-α	Tumor necrosis factor α
IL	Interleukin
GM-CSF	Granulocyte-macrophage colony-stimulating factor
TCR	T-cell receptor
Erk/MAPK	Extracellular signal-regulate kinase/mitogen-activated protein kinases
ECP	Eosinophilic cationic protein
ILC2s	Group 2 innate lymphoid cells
Tregs	Regulatory T cells
OPN	Osteopontin
AREG	Amphiregulin
IL-5R	IL-5 receptor
TSLP	Thymic stromal lymphopoietin
